# Supplementary material for: Oriented 3D Magnetic Biskyrmions in MnNiGa Bulk Crystals
Source: Adv Mater. Author manuscript; Available in PMC 2024 Jul 29. (PMC11284572; doi:10.1002/adma.201900264)
Supplement: suppl [file NIHMS1613545-supplement-suppl.pdf]

Copyright WILEY-VCH Verlag GmbH & Co. KGaA, 69469 Weinheim, Germany, 2019.

ADVANCED MATERIALS

Supporting Information

for *Adv. Mater.,* DOI: 10.1002/adma.201900264

Oriented 3D Magnetic Biskyrmions in MnNiGa Bulk Crystals

*Xiyang Li, Shilei Zhang, Hang Li, Diego Alba Venero, Jonathan S White, Robert Cubitt, Qingzhen Huang, Jie Chen, Lunhua He, Gerritvan der Laan, Wenhong Wang, Thorsten Hesjedal, and Fangwei Wang**

Supporting Information

Oriented Three-Dimensional Magnetic Biskyrmion in MnNiGa Bulk Crystals

Xiyang Li, Shilei Zhang, Hang Li, Diego Alba Venero, Jonathan S White, Robert Cubitt, Qingzhen Huang, Jie Chen, Lunhua He, Gerrit van der Laan, Wenhong Wang, Thorsten Hesjedal, and Fangwei Wang*

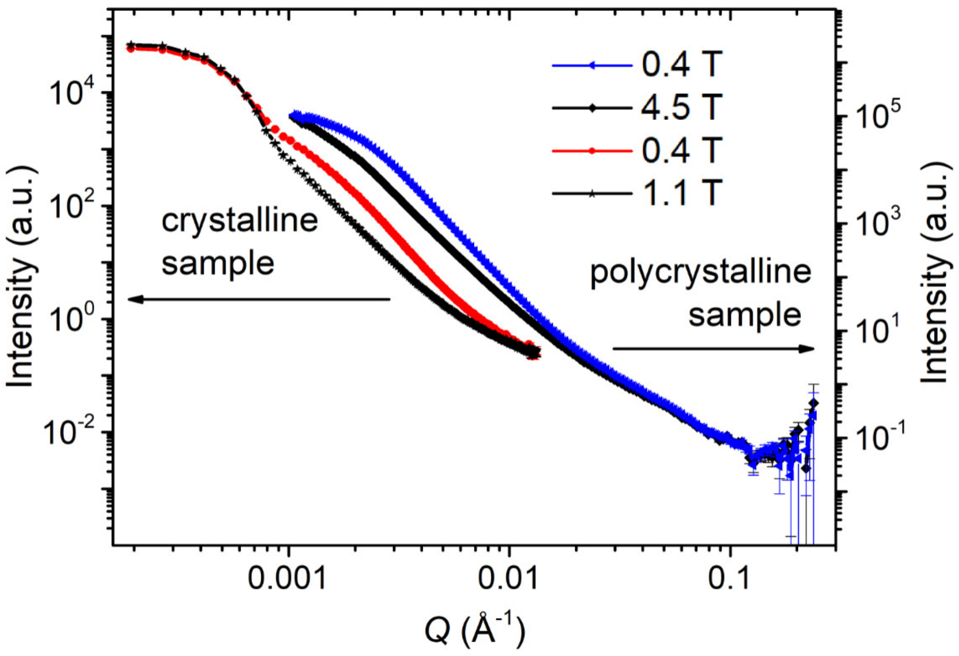

Figure S1: Reduced 1-dimensional data for the polycrystalline sample measured on SANS2d@ISIS and for the single-crystalline sample measured on D11@ILL. The scattering intensity for the low field biskyrmion state is larger compared to the high-field polarized state.

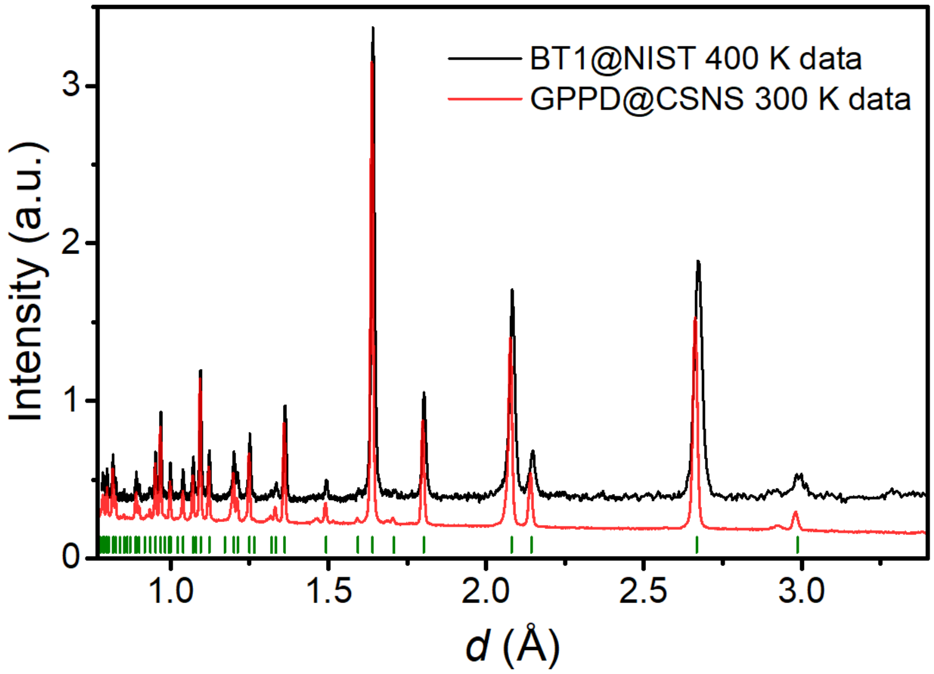

Figure S2: Comparison of neutron diffraction data obtained at 300 K and 400 K. The Curie temperature of the sample is ~350 K (as reported in reference [19]). Note that no magnetic satellites associated with the helical/conical phase are measured at 300 K as the helical period is too large to be detected by neutron diffraction. The green lines represent the Bragg peak positions of the crystalline structure.
